# A Digital Hardware System for Spiking Network of Tactile Afferents

**DOI:** 10.3389/fnins.2019.01330

**Published:** 2020-01-14

**Authors:** Nima Salimi-Nezhad, Erfan Ilbeigi, Mahmood Amiri, Egidio Falotico, Cecilia Laschi

**Affiliations:** ^1^Medical Biology Research Center, Kermanshah University of Medical Sciences, Kermanshah, Iran; ^2^Medical Technology Research Center, Kermanshah University of Medical Sciences, Kermanshah, Iran; ^3^The BioRobotics Institute, Scuola Superiore Sant’Anna, Pontedera, Italy

**Keywords:** tactile sensing, spiking network, digital circuit, mechanoreceptor, primary afferents

## Abstract

In the present research, we explore the possibility of utilizing a hardware-based neuromorphic approach to develop a tactile sensory system at the level of first-order afferents, which are slowly adapting type 1 (SA-I) and fast adapting type 1 (FA-I) afferents. Four spiking models are used to mimic neural signals of both SA-I and FA-I primary afferents. Next, a digital circuit is designed for each spiking model for both afferents to be implemented on the field-programmable gate array (FPGA). The four different digital circuits are then compared from source utilization point of view to find the minimum cost circuit for creating a population of digital afferents. In this way, the firing responses of both SA-I and FA-I afferents are physically measured in hardware. Finally, a population of 243 afferents consisting of 90 SA-I and 153 FA-I digital neuromorphic circuits are implemented on the FPGA. The FPGA also receives nine inputs from the force sensors through an interfacing board. Therefore, the data of multiple inputs are processed by the spiking network of tactile afferents, simultaneously. Benefiting from parallel processing capabilities of FPGA, the proposed architecture offers a low-cost neuromorphic structure for tactile information processing. Applying machine learning algorithms on the artificial spiking patterns collected from FPGA, we successfully classified three different objects based on the firing rate paradigm. Consequently, the proposed neuromorphic system provides the opportunity for development of new tactile processing component for robotic and prosthetic applications.

## Introduction

The sense of touch covers the whole body using a variety of receptors in different depth of skin. Information coming from muscles and tendons (kinesthetic sensing) and rich signals from touch receptors embedded in the skin (cutaneous sensing) play a crucial role in our sensory experience, and thus, we are able to actively communicate with our surrounding world. Specifically, when we interact with an object, information about that object characteristics such as its shape and texture is carried in the spatiotemporal pattern of action potentials evoked in a variety of tactile afferents. These action potentials or *spikes* are transmitted by the primary afferents to the spinal cord, cuneate nucleus, thalamus, and finally somatosensory cortex for decoding and decision making. Consequently, we are able to recognize objects based on tactile exploration (Dahiya et al., 2010, 2013). The specialized mechanoreceptors in the human glabrous skin are composed of two main types, based on their functionality and their receptive field, (1) slowly adapting (SA) afferent and (2) the fast adapting (FA) afferent (Dahiya and Valle, 2012; Tiwana et al., 2012). The SA type 1 (SA-I) and type II (SA-II) afferents innervate Merkel and Ruffini cylinder, respectively, and are mostly sensitive to static stimuli. The FA type 1 (FA-I) and type II (FA-II) afferents, which are sensitive to transient events such as vibration, innervate the Meissner corpuscle and Pacinian corpuscle, respectively (Lucarotti et al., 2013). In this study, we focus on the SA-I and FA-I tactile afferents, which are necessary elements for object manipulation (Johansson and Flanagan, 2009).

Recent approaches aim to mimic the behavior of the biological tactile receptors using advanced skin dynamics (Saal et al., 2017) and neuromorphic models (Oddo et al., 2016) to progress the efficiency and performance over traditional techniques. Application of spiking neural networks and neuromorphic approaches in tactile systems are increasing in the past few years (Kim et al., 2009; Friedl et al., 2016; Oddo et al., 2016; Yi and Zhang, 2016). Pearson et al. (2006, 2007, 2011) developed a biomimetic vibrotactile sensory system using leaky integrate-and-fire neuron models, which replicates rat whiskers for enabling a robot to navigate its environment. To discriminate local curvature of an object, Lee et al. (2013) used a fabric-based binary tactile sensor array. The tactile signals were converted into spikes using the Izhikevich model (Lee et al., 2014). For decoding Braille letters, a closed perception-action loop was made by converting force sensor data to spike trains using the leaky integrate-and-fire model (Bologna et al., 2011, 2013). An Izhikevich neuron model was used by Spigler et al. (2012) for characterizing surface properties. Zhengkun and Yilei (2017) transformed the outputs of polyvinylidene difluoride tactile sensors to spike trains using the Izhikevich model and then applied machine learning algorithm for classification of surface roughness. Rongala et al. (2017) classified 10 naturalistic textures by converting the outputs of an array of four piezoresistive sensors into spike trains. They used the Izhikevich model and analyzed the obtained spiking patterns (Rongala et al., 2017). Using the same sensor, Oddo et al. transduced haptic stimulus into a spatiotemporal pattern of spikes and then applied them to the rat skin afferents using stimulation electrodes. In this way, they showed a potential for neuro-prosthetic approach to communicate with the rat brain (Oddo et al., 2017). Moreover, neuromorphic techniques have been used to induce tactile sensation for differentiating textures using SA-like dynamics through nerve stimulation of an amputee (Oddo et al., 2016) and to enhance grip functionality of the prosthesis (Osborn et al., 2017). In Osborn et al. (2018), it was focused on pain detection through a neuromorphic interface and initiated an automated pain reflex in the prosthesis.

One of the most common methods to realize the neural computational models is developing digital circuit due to its high efficiency for practical applications (Cassidy et al., 2011). Digital execution with field-programmable gate array (FPGA) affords flexibility necessary for algorithm exploration while filling time and performance constraints. Therefore, FPGAs have increasing applications in the neural computing area (Nanami and Kohno, 2016). Furthermore, with the advancement in HDL (high-level hardware description language) synthesis tools, FPGA can also be operated as the effective hardware accelerators (Misra and Saha, 2010; Arthur et al., 2012). Some researchers have worked on efficient hardware implementations (Wang et al., 2018; Zjajo et al., 2018). Grassia et al. (2016) simulated a stochastic neuron in FPGA. An approximate circuit technique was used for FPGA implementation of real-time processing of tactile data to be utilized in the e-skin applications (Franceschi et al., 2017). Ambroise et al. (2017) proposed a biomimetic neural network implemented on FPGA for bi-directional communication with living neurons cultured in microelectrode array. A digital hardware realization was proposed for spiking model of cutaneous mechanoreceptor in order to identify the applied pressure stimulus (Salimi-Nezhad et al., 2018). They used the Izhikevich neuron model for simulation and then digital execution of the SA-I and FA-I afferents on the FPGA. Indeed, their approach is the proof of concept that digital circuit implementation of tactile afferents has great potential. However, it is necessary to extend previous work that considers one SA-I or FA-I digital circuit with single input. Actually, tactile information is conveyed not only using multiple sub-modalities but also through ensembles of different afferent types. Consequently, developing a hardware-based neuromorphic system to run a population of various afferents and receive multiple inputs is necessary for modeling study and fabrication of novel tactile sensory system for robotic and prosthetic applications. Accordingly, in this paper, we report that designing of a neuromorphic tactile system using a population of 243 digital afferents includes SA-I and FA-I. To this purpose, first, four spiking models including Izhikevich model (Izh), linearized Izhikevich model (L-Izh), Quadratic Integrated and Fire model (QIF), and linearized QIF model (L-QIF) are considered for simulating the neural afferents. Next, for all of these spiking models, an appropriate digital circuit is presented and simulated in VIVADO. The performance comparison is done to find which of the designed circuit is efficient from area and power consumption viewpoint while maintaining the characteristics of their original mathematical model. Then, the superior circuit is further improved by replacing multipliers with logical shifter. Consequently, the *improved* L-QIF was hired for each afferent to create a neuromorphic network of artificial SA-I/FA-I afferents. Employing an experimental setup, the performance of the digital spiking network, which is executed on the FPGA, is explored. In this case, the indentation data of a 3 × 3 pressure sensor grid are sent to the FPGA through an interface board. FPGA runs the digital circuits of the 243 spiking model of afferents and processes the incoming data of nine pressure sensors in parallel to deliver tactile spike patterns for the next level of processing. To the best our knowledge, the proposed neuromorphic system is the first digital system implementing a population of tactile afferents (both SA-I and FA-I) while receiving multiple inputs. Finally, by applying machine learning algorithm, the artificial spike responses are analyzed based on the firing rate paradigm, and thus, we classify three objects to show a real application of the proposed neuromorphic tactile system in a haptic experiment.

The rest of the paper are prepared in this way: the spiking models and their digital circuits are described in sections “Materials and Methods” and “Digital Circuits,” respectively. The hardware implementation results are discussed in section “Hardware Implementation.” Finally, the section “Conclusion” concludes the paper.

## Materials and Methods

The mathematical description of four spiking models used in this research has been explained in Appendix. Based on these spiking models, we present an appropriate digital circuit for each model. The designed digital circuits are compared to obtain the circuit with minimum area and power consumption characteristics to be used for developing a neuromorphic tactile system.

### Spiking Model of Tactile Afferent

The primary afferents in the glabrous skin that convey tactile information are SA-I, II and FA-I, II. In human hand, there are approximately 43% FA-I afferents end with Meissner corpuscles, 13% FA-II units with Pacinian endings, 25% SA-I units innervate Merkel cells, and 19% SA-II units with Ruffini endings (McGlone and Reilly, 2010). Merkel receptors, located superficially in the skin (Roudaut et al., 2012), are triggered by lower-frequency skin deformations and are essential for texture discrimination and fine tactile perception. The SA-I afferents, which branch and innervate the Merkel discs, are active throughout the physical stimulation. Meissner receptors have particularly high density on the fingertips and respond whenever a change in the stimuli is detected (i.e., when the stimuli is applied or when it is removed) (Roudaut et al., 2012). The FA-I afferents, which branch and innervate the Meissner corpuscles, have small receptive fields and detect dynamic skin deformations (Johansson and Vallbo, 1979). They are responsible for detection of low-frequency vibration, slip, and motion.

**FIGURE 1:**
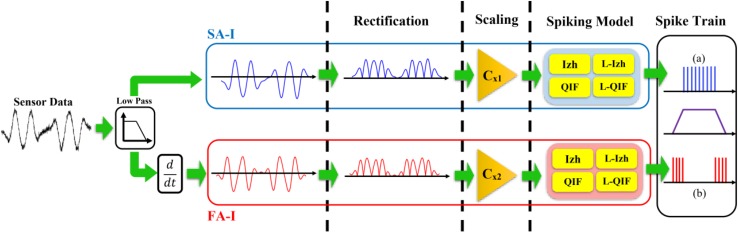
The slowly adapting type 1 (SA-I) and fast adapting type 1 (FA-I) afferents model. The SA-I responds to the absolute value of the stimulus and is active all over the interval of the stimulus contact. The FA-I delivers spikes when stimulus has dynamic, i.e., during onset and offset phases of indentation profile. Four neural models including the Izhikevich model (Izh), linearized Izhikevich model (L-Izh), Quadratic Integrated and Fire model (QIF), and linearized QIF model (L-QIF) are used for spike generation of the afferent model, independently.

Figure 1 shows the afferent model used in this study. It was shown that this model reproduces the spike trains generated in the FA-I and SA-I biological counterpart for various stimuli (Saal and Bensmaia, 2015; Friedl et al., 2016; Rongala et al., 2017, 2018; Salimi-Nezhad et al., 2018). In this model, the amount of force value is measured by the sensor, *f*(*t*) and its variations $\dot{\text{f}}$ (*t*) (in mN), are weighted separately (*C_x1_*, *C_x2_*) to make the current *I*(*t*) (in mA) for spike generation. Four neural models including Izh, L-Izh, QIF, and L-QIF are used for spiking part of the afferent model, independently. The mathematical descriptions of these four spiking models are explained in Appendix.

## Digital Circuits

For designing neuromorphic systems, FPGAs are frequently used in recent years, and several successful cases were reported in the literature. Indeed, its parallel and high-speed computation ability afford real-time implementation of spiking neural networks. In this section, spiking models are first discretized using Euler method, and then the digital circuits to be executed on FPGA are presented. For the designed digital circuits, the resource utilization is compared to find the circuit, which has fewer logic blocks. In this way, we can implement a large population of afferents. The discretizing step for all equations is *h* = 0.0078125 ms. In the following equations, we consider that *C*_*m*_ and τ are equal to 1 F and 1 s, respectively.

### The Izh Digital Circuit

Equations 21–23 describing the spiking behavior of the SA-I model can be discretized as:

(1) $$v\left[n+1\right]=v\left[n\right]+h\times (0.04\times v\left[n\right]\times v\left[n\right]+5\times v\left[n\right]+140-u\left[n\right]+C_{11}\times I\left[n\right])$$

(2) u⁢[n+1]=u⁢[n]+h×a×(b×v⁢[n]-u⁢[n])

(3) $$\text{if}\,v\,\left[n+1\right]\geq 30\,\text{mV}\to \mathrm{then}\,\left\{ \begin{array}{c} v\,\left[n+1\right]\leftarrow c\\ u\,\left[n+1\right]\leftarrow u\,\left[n\right]+d \end{array}\right.$$

**FIGURE 2:**
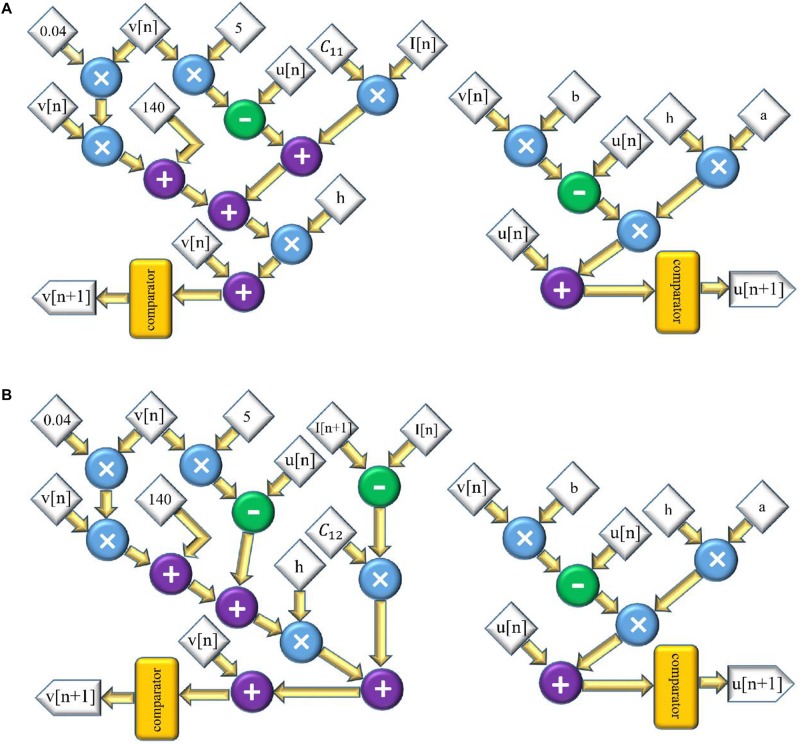
The scheduling diagram for spike generation of SA-I afferent **(A)** and FA-I afferent **(B)** using the Izh spiking model. In these diagrams, there are two state variables, *v* and *u*, so two digital circuits are designed for each variable, distinctly.

The scheduling diagram for this model is illustrated in Figure 2A. Similarly, for the FA-I model, the discretized equations are as follows:

(4) $$v\left[n+1\right]=v\left[n\right]+h\times (0.04\times v\left[n\right]\times v\left[n\right]+5\times v\left[n\right]+140-u\left[n\right])+C{}_{12}\times (I\left[n+1\right]-I\left[n\right])$$

(5) u⁢[n+1]=u⁢[n]+h×a×(b×v⁢[n]-u⁢[n])

(6) $$\text{if}\,v\,\left[n+1\right]\geq 30\,\text{mV}\to \text{then}\,\left\{ \begin{array}{c} v\,\left[n+1\right]\leftarrow c\\ u\,\left[n+1\right]\leftarrow u\,\left[n\right]+d \end{array}\right.$$

The scheduling diagram for the FA-I model is shown in Figure 2B. It illustrates how membrane potential (*v*) and recovery variable (*u*) of the afferent model in each iteration are generated. There are also memory registers to store the outputs for use in the subsequent steps. The register length, *N*, to solve individual state variables is *N* = 32 (1 bit for sign, 13 bits for integer part, and 18 bits for fractional part) to obtain a low-error and high-speed circuit (Salimi-Nezhad et al., 2018). It should be pointed out that “*N*” directly affects the computational time and the required precision for implementation.

### The L-Izh Digital Circuit

To design the digital circuit for the L-Izh model of the SA-I afferent, Eqs 27–29 are discretized as follows:

(7) $$v\left[n+1\right]=v\left[n\right]+h\times (k_{1}\times |v\left[n\right]+62.5|-k_{2}-u\left[n\right]+C_{21}\times I\left[n\right])$$

(8) u⁢[n+1]=u⁢[n]+h×a×(b×v⁢[n]-u⁢[n])

(9) $$\text{if}\,v\,\left[n+1\right]\geq 30\,\text{mV}\to \mathrm{then}\,\left\{ \begin{array}{c} v\,\left[n+1\right]\leftarrow c\\ u\,\left[n+1\right]\leftarrow u\,\left[n\right]+d \end{array}\right.$$

**FIGURE 3:**
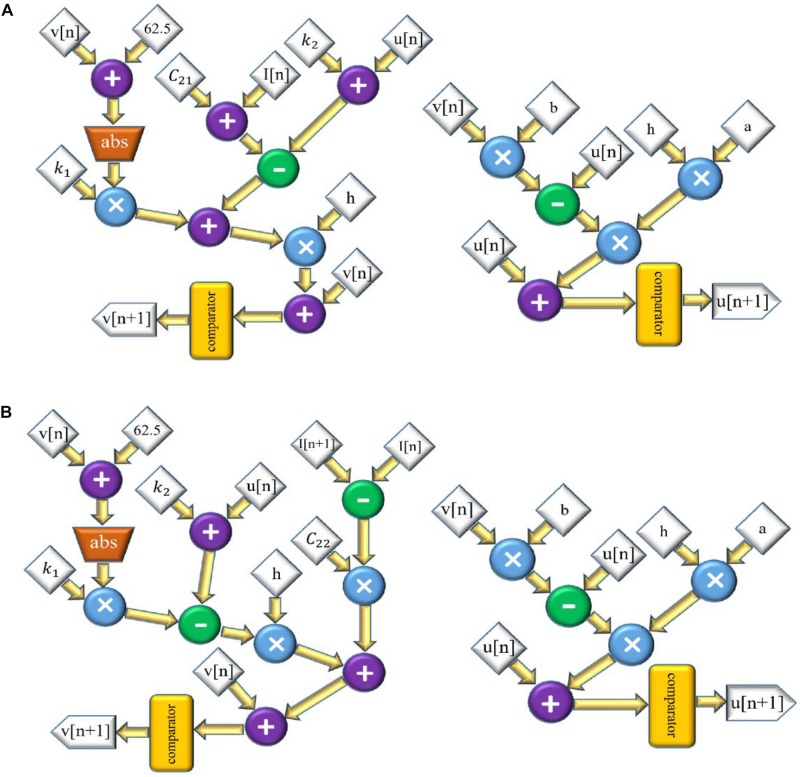
The scheduling diagram for spike generation of SA-I afferent **(A)** and FA-I afferent **(B)** using the L-Izh model. In these diagrams, there are two state variables, *v* and *u*, so two digital circuits are designed for each variable, separately. Compared to Figure 2, due to linearization, this model consumes less hardware area and has less power consumption.

Accordingly, the scheduling diagram is depicted in Figure 3A. For FA-I afferent, the discrete equations of L-Izh model are as follows:

(10) $$v\left[n+1\right]=v\left[n\right]+h\times \left(k_{1}\times |v\left[n\right]+62.5\right|-k_{2}-u\left[n\right])+C{}_{22}\times (I\left[n+1\right]-I\left[n\right])$$

(11) u⁢[n+1]=u⁢[n]+h×a×(b×v⁢[n]-u⁢[n])

(12) $$\text{if}\,v\,\left[n+1\right]\geq 30\,\text{mV}\to \text{then}\,\left\{ \begin{array}{c} v\,\left[n+1\right]\leftarrow c\\ u\,\left[n+1\right]\leftarrow u\,\left[n\right]+d \end{array}\right.$$

and the scheduling diagram is presented in Figure 3B. It shows how membrane potential (*v*) and recovery variable (*u*) of the afferent model in each iteration are produced.

### The QIF Digital Circuit

**FIGURE 4:**
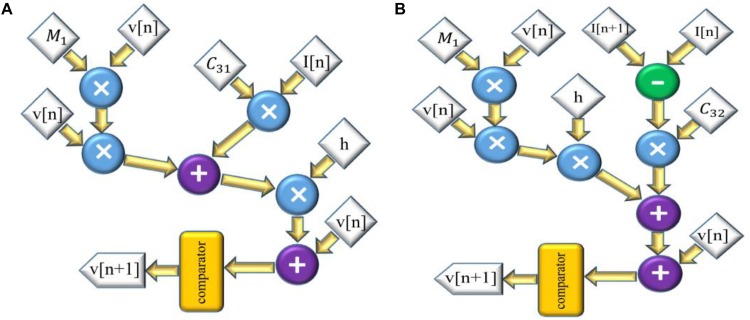
The scheduling diagram for spike generation of SA-I afferent **(A)** and FA-I afferent **(B)** using the QIF model. In these diagrams, there is only one state variable, *v*, the membrane potential. Compared to Figures 2, 3, the digital circuit of the QIF model is simpler, consumes less hardware area, and has less power consumption.

Equations (33)–(34), which are responsible for producing spiking patterns in the SA-I model, are discretized as follows:

(13) $$v\,\left[n+1\right]=v\,\left[n\right]+h\times \left(M_{1}\times v\,\left[n\right]\times v\,\left[n\right]+C_{31}\times I\,\left[n\right]\right)$$

(14) $$\text{if}\,v\,\left[n+1\right]\geq v_{\text{peak}}\to \mathrm{then}\,v\,\left[n+1\right]=v_{\text{reset}}$$

and the scheduling diagram for this model is presented in Figure 4A. Also, the discretized equations for FA-I model are:

(15) $$v\,\left[n+1\right]=v\,\left[n\right]+h\times \left(M_{1}\times v\,\left[n\right]\times v\,\left[n\right]\right)+C_{32}\times \left(I\,\left[n+1\right]-I\,\left[n\right]\right)$$

(16) $$\text{if}\,v\,\left[n+1\right]\geq v_{\text{peak}}\to \text{then}\,v\,\left[n+1\right]=v_{\text{reset}}$$

The scheduling diagram for this model is shown in Figure 4B.

### The L-QIF Digital Circuit

**FIGURE 5:**
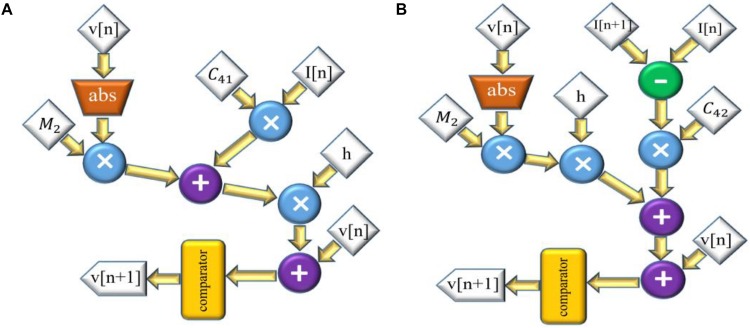
The scheduling diagram for spike generation of SA-I afferent **(A)** and FA-I afferent **(B)** using the L-QIF model. In these diagrams, there is only one state variable, *v*, the membrane potential. Compared to Figures 2–4, the digital circuit for the linearized version of the QIF model is much simpler, consumes less hardware area, and has less power consumption.

Parallel to the method used in the previous subsections, Eqs 37 and 38 for SA-I model are discretized as follows:

(17) $$v\,\left[n+1\right]=v\,\left[n\right]+h\times \left(M_{2}\times \left|v\,\left[n\right]\right|+C_{41}\times I\,\left[n\right]\right)$$

(18) $$\text{if}\,v\,\left[n+1\right]\geq v_{\text{peak}}\to \text{then}\,v\,\left[n+1\right]=v_{\text{reset}}$$

and the scheduling diagram for this model is demonstrated in Figure 5A. Finally, the discretized equations for FA-I model are:

(19) $$v\,\left[n+1\right]=v\,\left[n\right]+h\times \left(M_{2}\times \left|v\,\left[n\right]\right|\right)+C_{42}\times \left(I\,\left[n+1\right]-I\,\left[n\right]\right)$$

(20) $$\text{if}\,v\,\left[n+1\right]\geq v_{\text{peak}}\to t\,h\,e\,n\,v\,\left[n+1\right]=v_{\text{reset}}$$

and the scheduling diagram is illustrated in Figure 5B.

The digital circuits, Figures 2–5, based on spiking model of afferents, are the neuromorphic conversion of sensor output to spiking patterns conveying tactile information. Table 1 compares the resources utilized by the different digital circuits for both SA-I and FA-I models. As it is observed, the digital circuits for the linearized models (L-Izh and L-QIF) are more area efficient compared with their original counterparts (the Izh and QIF models). Also, it is apparent that the L-QIF digital circuit uses the minimum resources. Considering Table 1, the hardware resource utilization even for the Izh digital circuits compared to the circuits reported in Salimi-Nezhad et al. (2018) is decreased. Specifically, in the present research, for the Izh digital circuits, we have used less number of DSP for SA-I and FA-I afferents compared to the circuits reported in Salimi-Nezhad et al. (2018).

**TABLE 1:** Device utilization summary for the four designed digital circuits for both afferents.

	**Izh**		**L-Izh**		**QIF**		**L-QIF**		
	**SA-I**	**FA-I**	**SA-I**	**FA-I**	**SA-I**	**FA-I**	**SA-I**	**FA-I**	**Available**
Slice LUTs	1341	1436	1098	1192	839	882	250	308	53,200
Slice registers	97	129	97	129	65	97	65	97	106,400
Slice	726	750	558	582	380	396	223	246	13,300
LUT flip flop pairs	34	34	34	34	18	18	18	18	53,200
DSP48E1	8	8	6	6	4	4	3	3	220

*Izh, Izhikevich model; L-Izh, linearized Izhikevich model; QIF, Quadratic Integrated and Fire model; L-QIF, linearized QIF model; SA-I, slowly adapting type 1; FA-I, fast adapting type 1.*

### Simulation Results

**FIGURE 6:**
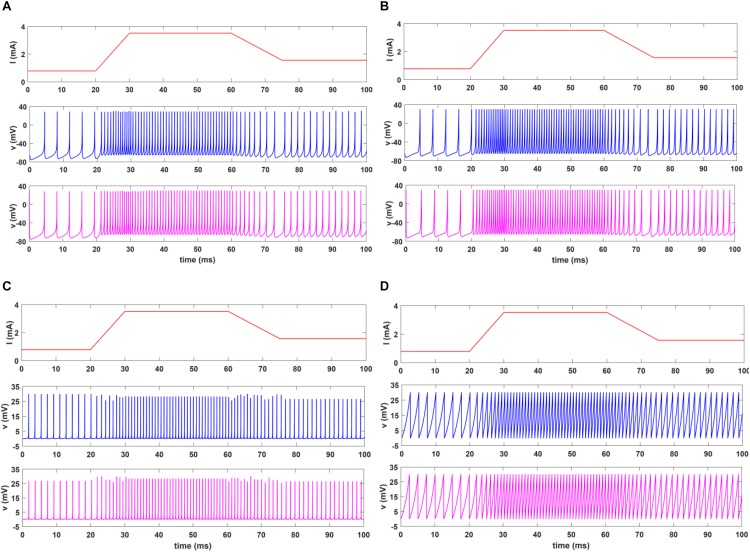
The time response of the spiking model of the SA-I afferent in 100 ms for Izh **(A)**, L-Izh **(B)**, QIF **(C)**, and L-QIF **(D)**. In these simulations, the first panels show the input signal, the second panels display the MATLAB simulation of the mathematical model, and the third panels illustrate the VIVADO simulation of the digital circuit. Average frequencies in 100-ms simulation for Izh, L-Izh, QIF, and L-QIF are 760, 840, 880, and 780 Hz, respectively.

**FIGURE 7:**
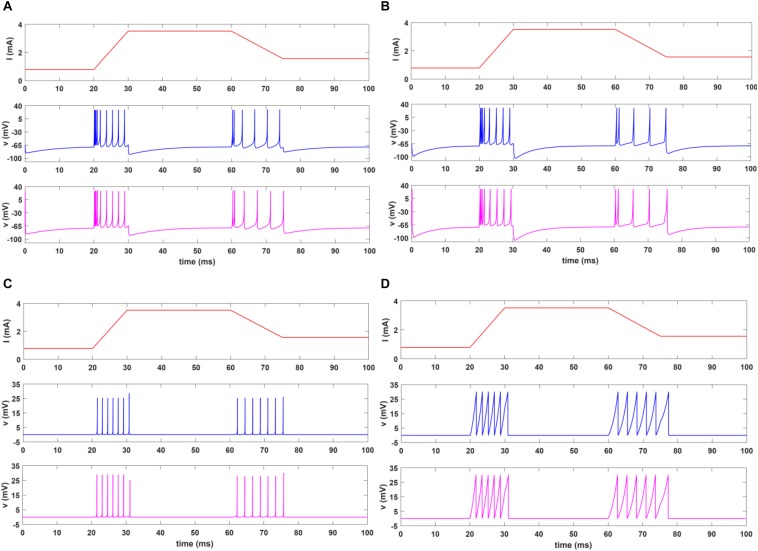
The time response of the spiking model of the FA-I afferent in 100 ms for Izh **(A)**, L-Izh **(B)**, QIF **(C)**, and L-QIF **(D)**. In these simulations, the first panels show the input signal, the second panels display the MATLAB simulation of the mathematical model, and the third panels illustrate the VIVADO simulation of the digital circuit.

In this section, we present the results of MATLAB simulation of four types of spiking models for both afferents (SA-I, FA-I) and VIVADO simulations of their digital circuits. Figure 6 shows the time responses of the SA-I spiking model with trapezoidal input. Increasing input current causes decreasing inter-spike interval. Figure 7 demonstrates the time responses of the FA-I spiking model with trapezoidal input. Higher value of slope motivates the model to produce spike patterns with higher rate. In Figures 6, 7, the first panels display the trapezoidal pulse as the input signal, the second panels present the MATLAB simulations of the afferent model, and the third panels demonstrate the VIVADO simulation of the digital circuit.

Considering Figures 6, 7, the SA-I afferent fires throughout a sustained phase of stimulus and the FA-I afferent responds at the onset and offset phases of that stimulus. This result is functionally in agreement with the response measured by the observations reported in Jörntell et al. (2014). In other words, the spiking model and their digital circuit have similar responses and functionally are compatible with spiking activity of biological afferent.

### Population of Digital Afferents

Although in previous sections, we found that the L-QIF model has the least area consumption in comparison with the other three models, we can also use other techniques for further reduction in the hardware utilization. Indeed, multipliers are costly blocks, which consume more power and use more area compared to the simple blocks such as adders or shifters. For this reason, by replacing multipliers with logical shifter, the *improved* L-QIF is obtained with the coefficients described in Table 2. Consequently, we expect an increase in operational frequency, due to the lack of high-cost operation (multipliers) to slow down the important paths. Furthermore, this approach reimburses the limited number of available multipliers on the chip and supports the implementation of larger spiking networks on the FPGA. Parameter values in Table 2 are chosen to show a better and a clear view of the spiking responses in the raster plot of population of afferents. In this way, we modified and tuned the experimental parameters from the simulation parameters.

**TABLE 2:** Parameter values of the improved L-QIF digital circuit.

*M*_2_	0.25
*C*_41_	0.5
*C*_42_	16

Table 3 compares the *improved* L-QIF digital circuit with the L-QIF circuit. It is apparent that replacing multipliers with shift registers leads to the decrease in DSP and Slice LUTs count, while the number of LUT Flip Flop increases. In this way, more resources can be saved if one uses the improved L-QIF model for spiking model of afferents. This can be quite important when a population of afferents is implemented on the FPGA. It should be pointed out that, although modern FPGAs have a significant number of DSP slices, equipping prosthesis and robotic hands with human-like skin needs implementation of thousands of mechanoreceptors and afferents to enable simultaneous transmission of tactile information. Therefore, saving energy and area utilization is quite important for practical applications. Here, we demonstrate a prototype for 243 artificial afferents that transmit spikes asynchronously conveying spatiotemporal features necessary for tactile perception.

**TABLE 3:** Comparison of the hardware utilization for the L-QIF and the improved L-QIF digital circuits for both afferents.

	**L-QIF**		**Improved L-QIF**		
	**SA-I**	**FA-I**	**SA-I**	**FA-I**	**Available**
Slice LUTs	250	308	108	167	53,200
Slice registers	65	97	32	64	106,400
Slice	223	246	30	56	13,300
LUT flip flop pairs	18	18	29	32	53,200
DSP48E1	3	3	0	0	220

## Hardware Implementation

**FIGURE 8:**
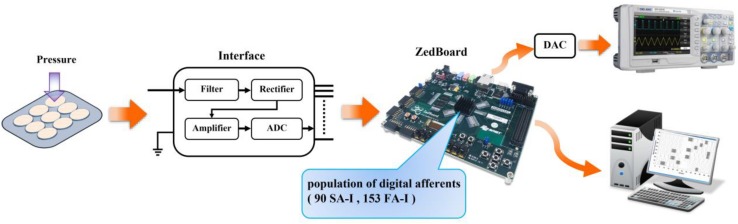
The experimental setup for evaluating the neuromorphic tactile system. A population of 90 digital SA-I afferents and 153 digital FA-I afferents are implemented on the field-programmable gate array (FPGA). In addition to the ZYNQ evaluation board, the system is composed of two other components: a matrix of 3 × 3 sensing units and an interface circuit (equipped with a 10-bit ADC unit) between the sensing unit and the ZedBoard. The sensing unit is composed of nine Force-Sensitive Resistors (FSRs) to deliver the detected pressure as an analog voltage signal to the interface unit. This unit filters, rectifies, and scales its input signal and then converts it to the digital signal to be sent to the ZedBoard. Resistance of the FSRs changes by applying an external force. Depending on the amount of pressure applied to the individual FSR sensor, digital afferents send spike trains to the screen or to the output pin of the ZedBoard to be displayed on the oscilloscope (after analog conversion).

The neuromorphic implementation of tactile afferents can speed up the development of novel artificial tactile sensory systems in the field of telerobotics and teleoperation. Consequently, in the current research, the hardware-based neuromorphic implementation is performed. To show the performance of the designed circuit and to illustrate the spiking patterns of a population of digital afferents, an experimental setup was developed as demonstrated in Figure 8. It consists of nine sensing units (a matrix of 3 × 3) connected to a ZedBoard through a custom interfacing board. The applied force to the individual Force-Sensitive Resistors (FSRs) provides an analog signal for the 10-bit ADC (analog to digital convertor), which is fed to the ZYNQ (in this case, ZedBoard). The ZedBoard (a particular ZYNQ evaluation board) is one of the low-cost and high-speed devices for digital realizations of spiking neurons. It is composed of two major sections: Programmable Logic (PL) and Processing System (PS). The PL section is a platform that can be configured using VHDL language and the PS section is a dual-core ARM cortex-A9 processor that can be programmed by C language. The output of ZedBoard is illustrated in two ways. One way is to show on oscilloscope, and the other is to display on the screen. Oscilloscope is used to show the spiking responses of the individual SA-I or FA-I digital circuit, and screen is employed to illustrate the activities of the whole population or subpopulation of digital afferents, simultaneously.

Due to resources available in the ZedBoard evaluation kit, we have implemented 243 digital circuits of the improved L-QIF models in the PL section including 90 SA-I and 153 FA-I. This ratio is chosen to consider that the number of SA-I and FA-I afferents exists in the fingertip (McGlone and Reilly, 2010; Pasluosta et al., 2017). In our design, for each FSR sensor, 27 digital afferents are run on the ZedBoard (10 SA-I and 17 FA-I). The hardware utilization for realization of 243 digital afferents is presented in Table 4. It should be mentioned that the operating frequency of ZYNQ is 100 MHz. Accordingly, in this experimental setup, the delay from the onset of applying force to the FSRs to the appearance of spiking responses on the ZYNQ output pins is in the range of nanoseconds.

**TABLE 4:** Hardware operation for realization of 243 afferents (SA-I/FA-I) in the ZedBoard using the improved L-QIF digital circuit.

	**Used**	**Available**
Slice LUTs	33,249(62%)	53,200
Slice registers	15,111(14%)	106,400
Slice	10,202(77%)	13,300
LUT as logic	33,187(62%)	53,200
LUT as memory	62(1%)	17,400
LUT flip flop pairs	8723(16%)	53,200
DSP48E1	180(82%)	220
Bonded IO	21(10%)	200

Considering final applications, simplicity of hardware implementation is an important factor. This feature is essential for development of sensory modules, which tries to integrate sensory and processing circuits. Indeed, spike-based representation of information has a significant potential to improve performance and efficiency of artificial tactile sensing systems. In this way, the proposed digital circuit enabled us to design a hardware architecture for executing a population of afferents on the PL. This new approach for fabricating sensory systems artificially replicates the firing patterns of the SA-I and FA-I afferents. The compartmentalized structure of the proposed approach and the ability to control parameters facilitate for easy scalability without extensive circuit redesign.

**FIGURE 9:**
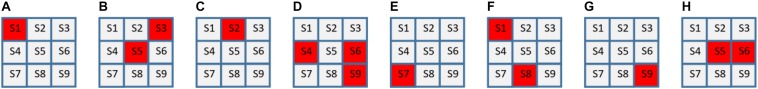
Randomly touching one **(A,C,E,G)**, two **(B,F,H)**, or three FSR sensors **(D)** from the 3 × 3 grid in the experimental setup shown in Figure 8. The activated sensors are shown by the red boxes. Different amounts of forces with dissimilar time profiles are applied to the FSR sensors. **(A)** to **(H)** show the sequence of touched sensors in eight stages, respectively.

Next, using the prepared experimental setup, we touch one, two, or three randomly selected FSR sensors simultaneously from the 3 × 3 pressure sensor matrix as illustrated in Figure 9. In this figure, the activated sensors are shown by the red boxes. For instance, in Figure 9D, three sensors are simultaneously touched, while in Figures 9B,F,H, two randomly selected sensors are touched at the same time.

**FIGURE 10:**
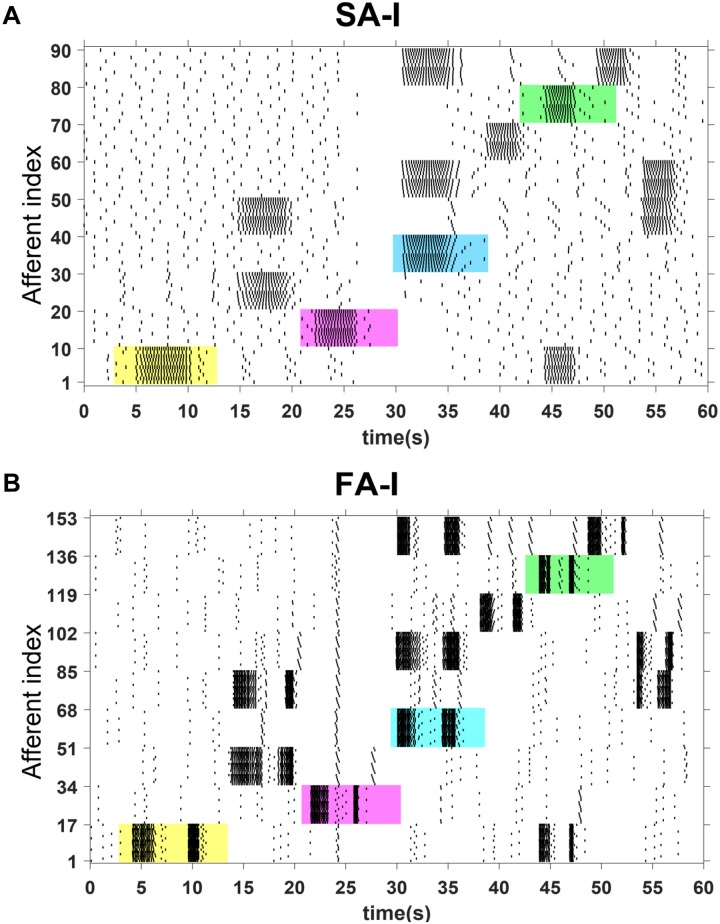
The raster plot for firing patterns of population of 243 digital afferents executed on the ZedBoard. The spiking patterns for 90 SA-I afferents **(A)** and 153 FA-I afferents **(B)** are shown for 60 s. Not only the touched sensors are selected randomly, but also the time duration and the onset and offset velocities are different. The spiking responses of four cases were highlighted by the colored regions to be further investigated in Figures 11, 12.

The spiking responses of the touched sensors in Figure 9 are shown in Figure 10. Figure 10A shows the spiking activity of the all 90 SA-I digital circuits, and Figure 10B demonstrates the spiking patterns of the all 153 FA-I digital circuits. Indeed, we used the FPGA parallel processing capability for realizing population of digital afferents. In Figure 10, for the first 4 s, no sensor has been touched and only the background activity of the population of artificial afferents is observed. Next, regarding Figure 9A, the first sensor, S1, is touched. In this case, from *t* = 4 to *t* = 6 s, the applied force to S1 sensor increases from zero to a desired level. From *t* = 6 to *t* = 9.5 s, the force value is maintained in this level. From *t* = 9.5 to 10.5 s, the applied force is reduced to its initial value, which is zero. Figures 10A,B show the firing activities of the population of artificial afferents, which are running on the ZedBoard. The digital SA-I afferents remain active during the period of stimulus contact, while the digital FA-I afferents react whenever a change in the stimuli is detected. Similarly, considering Figure 9B, both sensors S3 and S5 are touched concurrently. In this way, from *t* = 14 to 17 s, the applied forces to S3 and S5 increase from zero to another chosen level. From *t* = 17 to 19 s, the force value is maintained in this selected level. From *t* = 19 to 20 s, the applied force is again reduced to its initial value, which is zero. It should be pointed out that the amount of applied force to S3 is higher than S5, and thus, firing rate is increased, accordingly. Regarding Figure 10, the firing rate of the artificial SA-I is proportional to the intensity of stimulus, while firing patterns of the artificial FA-I appear when there is a changes in the stimulus intensity. Indeed, the different spiking sequences are evoked by applying different force profiles to the FSR sensors.

**FIGURE 11:**
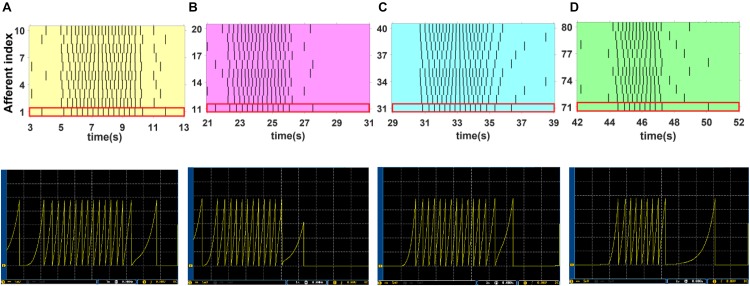
The firing activity of 10 digital SA-I afferents implemented on the ZedBoard. The sustained firing to the input is clear. Considering Figures 9, 10, yellow, magenta, cyan, and green illustrate the spiking activity arising from touching S1 **(A)**, S2 **(B)**, S4 **(C)** and S8 **(D)** respectively **(upper panels)**. We use a 16-bit DAC to convert the digital outputs of the ZedBoard to analog signals to be displayed on the oscilloscope screen **(lower panels)**. From each subpopulation, the first artificial afferent is chosen (the red rectangle in the upper panels) to be shown on the oscilloscope screen **(lower panels)**. The volt division was set on 5 mV.

**FIGURE 12:**
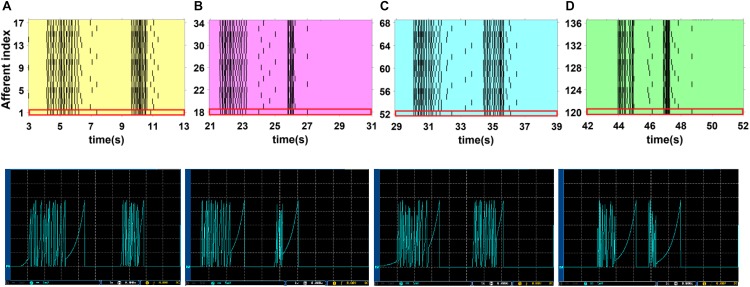
The firing activity of 17 digital FA-I afferents implemented on the ZedBoard. Considering Figures 9, 10, yellow, magenta, cyan, and green illustrate the spiking activity by touching S1 **(A)**, S2 **(B)**, S4 **(C)** and S8 **(D)**, respectively **(upper panels)**. Individual digital FA-I afferent fires during stimulus onset and offset and changes in input. We use a 16-bit DAC to convert the digital outputs of the ZedBoard to analog signals to be shown on the oscilloscope screen **(lower panels)**. From each subpopulation, the first artificial afferent is chosen (the red rectangle in the upper panels) to be displayed on the oscilloscope **(lower panels)**. The volt division was set on 5 mV.

To obtain more insights, we select four cases from Figure 10, the colored regions, and then explore the behavior of the SA-I and FA-I digital circuits in more detail as shown in Figures 11, 12, respectively. In other words, not only the firing patterns of the whole population are illustrated in Figure 10, but also we show the spiking responses of the selected afferent on the oscilloscope screen (Figures 11, 12). Yellow, magenta, cyan, and green illustrate the spiking patterns arising from touching S1, S2, S4, and S8, respectively (see Figure 9). In Figures 11, 12, from each subpopulation, one (the first) implemented artificial afferent is chosen (the red rectangle in the colored regions) to be displayed on the oscilloscope. In this case, the output of the selected digital afferent after converting to analog signal is demonstrated on the oscilloscope. In these figures, the output of the ZedBoard was shown in yellow color (membrane voltage). As it is observed, as the force magnitude increases, the firing frequency of the spiking patterns for digital SA-I is also increased. This approach makes possible to decode stimuli, while the tactile data are collected. Moreover, it is observed from Figure 12 that the rate of spiking responses in the offset phase is less than the onset phase for digital FA-I, due to the smaller slope for the offset phase. Indeed, the SA-I afferents provide encoding of pressure and FA-I afferents encode transient performance of the signal.

**FIGURE 13:**
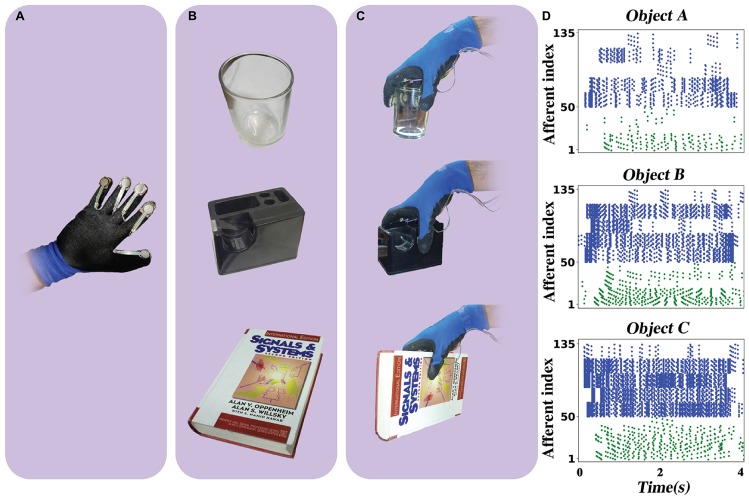
The haptic experiment. **(A)** Five FSR sensors are attached to a glove. **(B)** Three objects: a glass, a tape dispenser, and a book. **(C)** The subject wears the glove and picks up every object and holds it for 3 s and then puts in the place. The subject repeats this experiment for 20 times with three fingers, four fingers, and five fingers, independently. The FSRs send their signal to the ZedBoard where it runs a population of 50 SA-I and 85 FA-I digital afferents. The firing patterns of the 135 artificial tactile afferents are recorded for 180 trials (3 objects, 3 cases, 20 repetitions) to be analyzed by the machine learning algorithms. **(D)** Sample of spike trains with five fingers. Green spikes show the response of the artificial SA-I afferents and blue spikes illustrate the response of artificial FA-I afferents.

In addition, in order to show a practical application of the proposed neuromorphic setup, we attached five FSR sensors on a glove (each FSR sensor was allocated to a finger) and performed some haptic experiments while sending FSR outputs to the spiking network of tactile afferents implemented on the ZedBoard. The subject wears the glove to pick up, hold, and put in the place three different objects (a glass, a tape dispenser, and a book) while the firing activity of population of afferents is being measured. As shown in Figure 13, these objects have various size and weights. Object A, the glass, has the lowest weight, and object C, the book, is the heaviest one. Each experiment takes 4 s, and the hold phase is 3 s fixed. The subject accomplished the experiment for three cases: first by three fingers (thumb, index, and middle), then four fingers (thumb, index, middle, and ring), and finally with all five fingers. Each three-, four-, and five-finger experiment was done 20 times for individual objects. Consequently, 60 trials were collected for each object, and for every trial, firing responses of 50 digital SA-I and 85 FA-I afferents were recorded for 4 s from the ZedBoard. Indeed, the spiking patterns of the 135 artificial tactile afferents were recorded for 180 trials (3 objects, 3 cases, 20 repetitions) to be analyzed by the machine learning algorithms.

**FIGURE 14:**
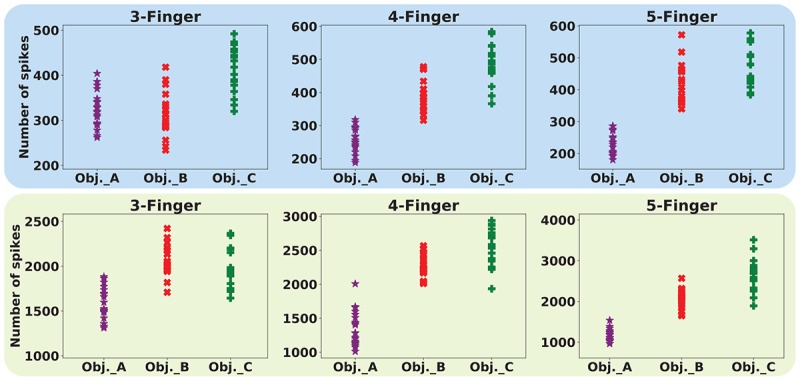
Decoding based on the firing rate paradigm, in which different stimuli elicit a different number of spikes for the same time interval. Upper panels and lower panels indicate spike count for SA-I and FA-I afferents, respectively. Each point indicates one trial. Twenty trials were performed for individual object, which is indicated by a different color.

**FIGURE 15:**
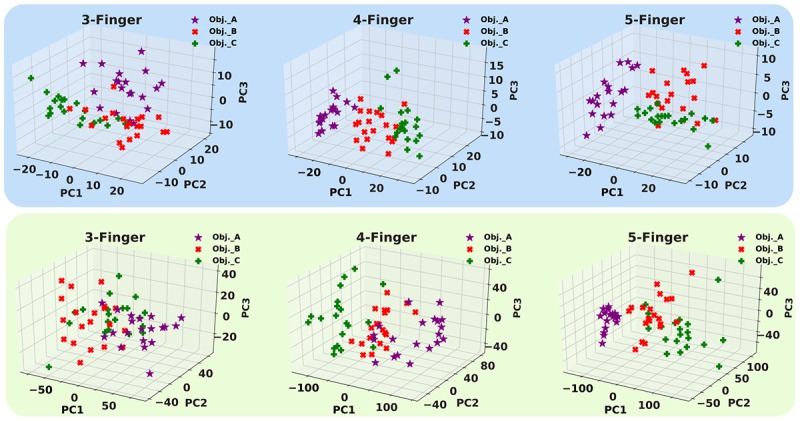
The first three principal components (PCs) obtained from three haptic experiments. Upper panels show feature space for SA-I afferents, and lower panels illustrate feature space for FA-I afferents.

Next, the machine learning approaches to interpret the recorded firing patterns are employed. In this way, first, feature extraction from spiking responses is accomplished using one of the fundamental coding paradigm for neural information processing, *rate coding.* The firing rate (FR) is defined by the number of spikes occurring at the time interval Δ*t*, *FR* = (*spikes*)/Δ*t*. Change of firing rate as the stimulus changes called *rate coding*. It is typically pointed out that sensory neurons transmit information by their firing rate. In this study, the decoding algorithm is based on the spike count; that is, different stimuli elicit a different number of spikes (Vreeken, 2003). Principal components analysis is exploited for dimension reduction. The first, three principal components are considered. Figure 14 shows the spike count of the population of SA-I and FA-I afferents for three objects and three cases. Each point indicates one trial. Feature space of the first three principle components for all three experiments is illustrated in Figure 15.

**FIGURE 16:**
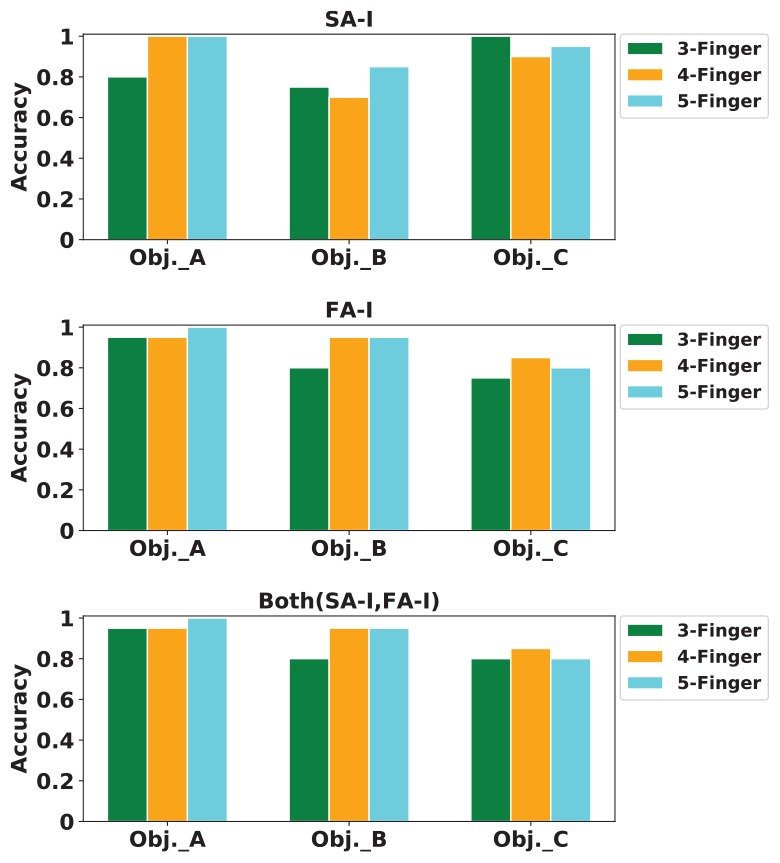
The classification accuracy for k-Nearest Neighbor (kNN) classifier. Classification accuracy for digital SA-I **(top panel)**, for digital FA-I **(middle panel)**, and for both afferents, SA-I and FA-I **(bottom panel)**.

Next, we report the classification performance of the k-Nearest Neighbor classifier using the obtained artificial spike trains. The classifier has three outputs: objects A, B, and C. The classifier input is the three principal components computed forming the total number of spikes obtained for that stimulus. Different *k* values from 2 to 8 were tried. However, the results for *k* = 5 were reported in Figure 16. The value of *k* is important as a small *k* might result to a classifier sensitive to noise samples, and a large *k* can lead to less distinct boundaries among classes. The k-Nearest Neighbor is a non-parametric classifier that measures the difference between every spike train (*ST*) and other spike trains. The object was properly classified when the mean difference between the *ST* and spike trains from the same class was smaller than the mean difference between the *ST* and spike trains of other classes. This procedure was repeated for every ST obtained from digital afferents.

For classification, 80% of samples were randomly grouped as training set, and the remaining 20% samples were considered as the test set. K-fold cross-validation was also used. Indeed, the data samples are divided into *K* subsets. Each time, one of these *K* subsets is used as the validation set and the remaining (*K* - 1) subsets form a training set. Then, the average error across all *K* trials for each subset is computed (Hosseini et al., 2007). We used *K* = 5 for cross-validation. The feature vectors must be normalized in order to avoid distortions between features and numerical problems. Finally, the mean and SD of classification accuracy for this haptic experiment (Figure 13C) is reported in Table 5.

**TABLE 5:** Mean and SD of classification accuracy for different experiment.

	**3-Finger**	**4-Finger**	**5-Finger**
SA-I	85% ± 3%	87% ± 8%	93% ± 6%
FA-I	83% ± 5%	92% ± 7%	92% ± 7%
Both (SA-I and FA-I)	85% ± 6%	92% ± 7%	92% ± 7%

**TABLE 6:** Parameter values for the spiking models of the tactile afferents used for simulations.

**Parameter**	**Value**	**Parameter**	**Value**
*a*	0.02 s^–1^	*b*	0.2 s^–1^
*c*	−65 mV	*d*	8 mV
*k*_1_	0.75 s^–1^	*k*_2_	20 mV s^–1^
*M*_1_	1 mV^–1^ s^–1^	*M*_2_	0.0625 s^–1^
*v*_*peak*_	30 mV	*v*_*reset*_	0 mV
*C*_*m*_	1 F	τ	1 s
*C*_11_	20	*C*_12_	960
*C*_21_	24	*C*_22_	960
*C*_31_	0.015625	*C*_32_	0.5
*C*_41_	1	*C*_42_	40

The developed system makes it possible to encode force information by a sequence of spikes, mimicking the neural dynamics of SA-I and FA-I afferents. Indeed, the recorded artificial spike trains from ZedBoard, which runs the SA-I/FA-I digital circuits, carry sufficient information. In this way, the input stimulus is discriminated even using a commercial FSR sensor. This technical approach is an innovative one for manufacturing sensory systems that artificially replicate the SA-I and FA-I firing activities to be employed in the bio-robotic and prosthetic application. The obtained spike trains are diverse and reliable enough to be able to decode the presented stimuli with high accuracy.

## Conclusion

To obtain better performance and efficacy over traditional methods, recently, there is a tendency toward creating neuromorphic devices to mimic the biological systems. Software simulation and hardware realization of the SA-I and FA-I afferents might be considered as the neuromorphic approaches for restoring tactile feedback in upper limb prostheses. This methodology transmits tactile information more efficient, very similar to the healthy peripheral nervous system, to the next level, which can be the prosthesis controller. In this research, to digitally realize a population of 243 tactile afferents (90 SA-I and 153 FA-I) on FPGA, with emphasis on real-time functionality, a digital circuit was designed using an improved version of the L-QIF neural model. This model has been selected for the highest simplicity and lowest resource consumption of hardware implementation compared to the other model reported in this research. Using an experimental setup, we investigated the performance of the neuromorphic tactile system (comprising the SA-I and FA-I afferents) when it received multiple inputs simultaneously. Using a glove equipped with FSRs, we performed some haptic experiments and then we analyzed the spiking responses measured from the ZedBoard. Applying machine learning algorithm and considering firing rate coding, the picked up object was recognized with high accuracy from the recorded spike trains produced by the artificial tactile afferents.

Although we did not discuss the biological plausibility of the designed digital circuits, it was shown that they functionally follow the physiological observation, which is a basic step for moving forward. It should be mentioned that, whereas FSR transducers are integrated relatively easily with peripheral hardware and software, their application for mimicking mechanoreceptor response is not precise. In addition, a compliant skin-like layer should cover the FSR sensors. Finally, implementing a population of digital afferents might support the possibility for future development of new generation of tactile modules for prosthetic hands to reestablish sensory feedback for amputee. Moreover, the obtained spike trains from digital afferents may be further processed by the next level, which also can be done in hardware. This will make a neuromorphic sensory system for a mobile robot to accomplish various real-world tasks such as texture discrimination and object recognition.

## Data Availability Statement

The datasets generated for this study are available on request to the corresponding author.

## Author Contributions

NS-N, EI, MA, EF, and CL did conception, design and interpretation of the data, and drafting and revising the manuscript. NS, EI, and MA performed the experiments, acquired the data, and analyzed the data.

## Conflict of Interest

The authors declare that the research was conducted in the absence of any commercial or financial relationships that could be construed as a potential conflict of interest.

## Appendix

### Izhikevich Neuron Model (Izh)

Integrate-and-fire (IF) neuron models are popular and simple to simulate, which help to be used in large network computational studies; however, they lack physiological interpretability. In contrast, conductance-based models with high biophysical realism are expensive to simulate since they often have high-dimensional non-linear differential equations. They require the tuning of many parameters and thus preventing their use in large networks.

Izhikevich proposed a model for spiking neuron that combines the response diversity of conductance-based models and the computational efficiency of IF neurons. The Izhikevich model (Izh) is described as follows (Izhikevich, 2003):

(21) $$v^{\prime }=0.04\,v^{2}+5\,v+140-u+C_{11}\,\frac{I}{C_{m}}$$

(22) $$u^{\prime }=a\,\left(b\,v-u\right)$$

(23) $$\text{if}\,v\geq 30\,\text{mV}\to \text{then}\,\left\{ \begin{array}{c} v\leftarrow c\\ u\leftarrow u+d \end{array}\right.$$

*v* is the membrane potential of the neuron, *I* is the input current, and *u* is the membrane recovery variable. Constants *a*, *b*, *c*, and *d* are the neuron parameters. *C_11_* scales the input current. *C*_*m*_ is capacitance value for dimensionality consistency. The parameters values of the Izh model, which were used in this research, are listed in Table 6.

Equations 21–23 are used to describe the spiking part of the SA-I model. Similarly, for FA-I model, the following mathematical model is used to obtain the output spike train.

(24) $$v^{\prime }=0.04\,v^{2}+5\,v+140-u+C_{12}\,\frac{\tau }{C_{m}}\,I^{\prime }$$

(25) $$u^{\prime }=a\,\left(b\,v-u\right)$$

(26) $$\text{if}\,v\geq 30\,\text{mV}\to \mathrm{then}\,\left\{ \begin{array}{c} v\leftarrow c\\ u\leftarrow u+d \end{array}\right.$$

*C_2_* is a constant factor that scales the input and τ is the time constant, and their values were reported in Table 6.

### Linearized Izhikevich Neuron Model (L-Izh)

One solution to reduce high-cost mathematical operations is *linearization*. We use a piecewise-linear approximation of the Izh, which was presented in Soleimani et al. (2012). This L-Izh is described as follows:

(27) $$v^{\prime }=k_{1}\,\left|v+62.5\right|-k_{2}-u+C_{21}\,\frac{I}{C_{m}}$$

(28) $$u^{\prime }=a\,\left(b\,v-u\right)$$

(29) $$\text{if}\,v\geq 30\,\text{mV}\to \text{then}\,\left\{ \begin{array}{c} v\leftarrow c\\ u\leftarrow u+d \end{array}\right.$$

*k_1_* and *k_2_* are the constant values of the linearized model. *C_21_* scales the neuron input. For the FA-I model, the L-Izh model can be used as follows:

(30) $$v^{\prime }=k_{1}\,\left|v+62.5\right|-k_{2}-u+C_{22}\,\frac{\tau }{C_{m}}\,I^{\prime }$$

(31) $$u^{\prime }=a\,\left(b\,v-u\right)$$

(32) $$\text{if}\,v\geq 30\,\text{mV}\to \text{then }\,\left\{ \begin{array}{c} v\leftarrow c\\ u\leftarrow u+d \end{array}\right.$$

*C_22_* is the constant coefficient for scaling the input current. The parameter values are listed in Table 6.

### Quadratic Integrated & Fire Neuron Model (QIF)

Considerable research has been devoted to combine the economy of IF models with the physiological interpretability of conductance-based models. An example is the Quadratic Integrated and Fire (QIF) model that has the interpretation of a mathematical reduction of the conductance-based model of Hodgkin and Huxley (1952) (Van Pottelbergh et al., 2018). Several generalizations of the QIF model have been studied in the literature. Shlizerman and Holmes (2012) presented the QIF model with minimum computations as follows:

(33) $$v^{\prime }=M_{1}\,v^{2}+C_{31}\,\frac{I}{C_{m}}$$

(34) $$\text{if}\,v\geq v_{\text{peak}}\to \text{then}\,v=v_{\text{reset}}$$

*M_1_* and *C_31_* are the constant coefficients. *v*_*peak*_ is the maximum value of membrane voltage, and *v*_*reset*_ is the rest membrane potential. All these parameters are reported in Table 6. Similarly, for FA-I model, we have:

(35) $$v^{\prime }=M_{1}\,v^{2}+C_{32}\,\frac{\tau }{C_{m}}\,I^{\prime }$$

(36) $$\text{if}\,v\geq v_{\text{peak}}\to \text{then}\,v=v_{\text{reset}}$$

*C_32_* scales the model input. Some neuron models such as the QIF model (Benjamin et al., 2014) and adaptive exponential integrate and fire model (AdEx model) (Brette and Gerstner, 2005) do not instantly produce a spike. These neuron models hire alternative non-instant functions, which control the membrane potential once it reaches the threshold voltage (Lee et al., 2018).

### Linearized QIF Neuron Model (L-QIF)

Although the QIF model is a simple model, it is possible to use the linear approximation method to obtain a simpler model. Similar to the method used in Soleimani et al. (2012), the QIF model is linearized as follows for SA-I model:

(37) $$v^{\prime }=M_{2}\,\left|v\right|+C_{41}\,\frac{I}{C_{m}}$$

(38) $$\text{if}\,v\geq v_{\text{peak}}\to \text{then}\,v=v_{\text{reset}}$$

*M_2_* and *C_41_* are the constant coefficients. The linearized version of the QIF model for FA-I afferent is as follows:

(39) $$v^{\prime }=M_{2}\,\left|v\right|+C_{42}\,\frac{\tau }{C_{m}}\,I^{\prime }$$

(40) $$\text{if}\,v\geq v_{\text{peak}}\to \text{then}\,v=v_{\text{reset}}$$

*C_42_* is the constant parameter. The parameter values reported in Table 6 are taken from Izhikevich (2003); Shlizerman and Holmes (2012), Soleimani et al. (2012), and Rongala et al. (2018). The M2 value is selected to have minimum mean square error between the QIF spiking model and its linearized version. The parameters in last four rows adjusted by testing various values to obtain appropriate firing rate. Overall, high gain value causes a strong firing rate independent from the stimulus strength, and thus, the temporal structure of spikes is less informative. Conversely, low gain factors initiate low firing rate and accordingly a long latency in spike responses (Oddo et al., 2017). So, it is necessary to have a proper tradeoff.
